# Expiratory Muscle Strength Training to Improve Voice and Respiratory Outcomes After Laryngectomy: A Feasibility Study

**DOI:** 10.1111/1460-6984.70107

**Published:** 2025-08-15

**Authors:** Freya Sparks, Margaret Coffey, Lucy Dipper, Jessica Crowther, Simon Hamilton, Louise Occomore‐Kent, Katerina Hilari

**Affiliations:** ^1^ Department of Language and Communication Sciences School of Health and Medical Sciences City St George's, University of London London UK; ^2^ Barts Health NHS Trust London UK; ^3^ Imperial College Academic Health Science Centre London UK

**Keywords:** EMST, feasibility, laryngectomy, tracheoesophageal, voice

## Abstract

**Background:**

People with laryngectomy who use a tracheoesophageal voice prosthesis for communication experience changes to respiratory function resulting in reduced breath support and increased secretions. This impacts tracheoesophageal voice quality and volume. Expiratory muscle strength training (EMST) is an effective treatment for cough management, voice and respiratory function in other clinical populations, such as neurodegenerative conditions and benign voice disorders. There is limited evidence for the use of EMST in people with laryngectomy. This study explores EMST feasibility and preliminary efficacy with tracheoesophageal speakers. It is hypothesised that, like in other disorders, EMST can improve respiratory parameters and thus tracheoesophageal voice quality.

**Aims:**

(1) To investigate the feasibility and acceptability of EMST after laryngectomy. (2) To determine the impact of EMST on respiratory function; participant‐reported outcome measures (PROMS) of cough and secretion burden, voice‐related quality of life and experiences of communication; objective and perceptual measures of tracheoesophageal voice quality.

**Methods and Procedures:**

This was a before–after study with time (double baseline) providing experimental control. Participants completed a 5‐week EMST protocol and 6‐week maintenance period. Assessments were taken at two baselines, post‐EMST and at 6‐week follow‐up: respiratory: maximum expiratory pressure (MEP), peak flow, forced expiratory volume (FEV1); PROMS: CASA‐Q, SECEL, V‐RQOL; voice: voice recording for perceptual (clinician‐rated) analysis, maximum phonation time, decibels. One‐way repeated measures ANOVA or Friedman tests were used as appropriate to explore the pattern of change. Effect sizes were reported.

**Outcomes and Results:**

Ten participants (eight male and two female) completed EMST. Participants found EMST acceptable and beneficial, reporting subjective changes in breathing and exercise tolerance. There were no significant differences in any measures between the two baselines. Significant increase from baseline was found in peak flow, FEV1 and decibels post‐EMST training and post 6‐week maintenance period, respectively. MEP increased significantly from baseline to post‐EMST training only. PROMS showed a reduction in cough impact, sputum symptoms and reduced self‐evaluation of communication impairment post‐EMST. There was a non‐significant reduction in maximum phonation time and no significant differences in clinician‐rated voice quality post‐EMST training and maintenance periods. Participants required device modifications to enable safe usage of the device and to achieve an airtight connection at the neck stoma.

**Conclusions and Implications:**

EMST is an acceptable treatment for tracheoesophageal speakers; however, device modifications are required to support usage and minimise airway safety risks. Preliminary efficacy data demonstrate that EMST may improve parameters of respiratory function, volume and self‐perception of communication in PWL. A larger‐scale randomised trial is required to increase the robustness of findings. It is recommended that device manufacturers develop adapters to facilitate airtight connection to neck stomas and to increase device safety in the laryngectomy population.

**WHAT THIS PAPER ADDS:**

*What is already known on this subject*

- According to existing evidence, EMST is an effective treatment for improving cough strength and respiratory function. However, knowledge on the use of EMST after laryngectomy is limited, with only one existing study in evidence (Van Sluis et al. 2020).

*What this paper adds to existing knowledge*

- This innovative feasibility study provides evidence on the feasibility and acceptability of EMST with people with laryngectomy and preliminary data on its impact on perceptual and objective measures of tracheoesophageal voice, cough and secretion clearance.

*What are the potential or actual clinical implications of this work?*

- Subject to further testing, EMST may be an appropriate treatment to consider improving respiratory function, secretion clearance, vocal loudness and self‐perception of communication for tracheoesophageal speakers.

## 1 Introduction

After laryngectomy, airway secretion clearance is compromised by changes to the airway anatomy that reduce humidification. Air is inhaled directly via the neck stoma; therefore, the nose and mouth are bypassed, preventing filtering, warming and humidifying of the inhaled air. Air entering the trachea is colder and drier (Merol et al. 2012); and mucociliary clearance, that is, respiratory tract clearance through coordinated mucous movement propelled by cilia, is negatively impacted (Kelly et al. 2021). This leads to principal complaints of increased mucus production, the need for coughing and frequent forced expiration to effect airway clearance (Hilgers et al. 1990).  Cough strength is therefore an important function for PWL to enable airway clearance.

In normal anatomy, the cough mechanism is enacted in three phases, inspiratory, compressive and expiratory (Chang 2005). First, an inspiratory breath is taken, increasing lung volume to prepare for expiratory airflow. During the compressive phase, the vocal folds close and the expiratory muscles contract to build intrathoracic pressure. As the vocal folds open, expiratory airflow is released through compressed airways at high velocity (McCool 2006). The force of high velocity airflow aerosolises and removes material from the airway (Pitts et al. 2009), clearing material which cannot be eliminated by normal mucociliary transport (Bustamente‐Marin and Ostrowski 2017). Respiratory muscle strength is therefore a key component of an effective cough and, in turn, of airway clearance.

After laryngectomy, cough mechanics and respiratory function are altered due to the anatomical changes arising from the surgery. With the removal of the vocal folds, the compressive phase of the cough mechanism is disrupted as there is no glottic closure to build intrathoracic pressure. Cough is therefore less efficient and reliant on the inspiratory and expiratory phases, which impacts the ability to clear airway secretions (Yue et al. 2021).

Expiratory muscle strength training (EMST) involves breathing into a hand‐held device against resistance. The device contains a spring‐controlled valve that opens in response to sufficient pressure generated from an expiratory breath. The spring within the device is adjustable, allowing for an increase or decrease in the opening pressure. On generating a forceful expiration into the device, the expiratory muscles are engaged. Targeting maximum expiratory pressure (MEP), therefore, results in the ability to generate increased expiratory force, which in turn improves cough strength as a key component of the cough mechanism (Pitts et al. 2009). Where MEP is reduced, voluntary cough strength is diminished (Chiara et al. 2006).

EMST is found to increase MEP (Templeman and Roberts 2020) and cough function in healthy, elderly adults (Kim et al. 2008), neurodegenerative diseases (Chiara et al. 2006; Laciuga et al. 2014; Tawara et al. 2018) and after partial laryngectomy (Palmer et al. 2019). EMST is also found to increase MEP post‐radiation for head and neck cancer (Hutcheson et al., 2018) and in people with laryngeal voice disorders (Tsai et al. 2016).

Changes to respiratory function after laryngectomy also impact the ability to produce an adequate, functional tracheoesophageal voice. Reduced breath support can cause reduced words per breath (Lundstrom and Hammarberg 2011), reduced loudness (Ng and Woo 2021), and the presence of secretions in the pharyngoesophageal segment impacts the perceptual quality and intelligibility of the voice (Búa et al. 2017). Respiration during speech is doubly impacted by air leak at the tracheostoma and increased airflow resistance to phonation (Ward et al. 2007). Air leak occurs if there is a poor seal of the baseplate or laryngectomy tube to the peristomal skin, resulting in air escape during occlusion of the tracheostoma for voicing. Increased airflow resistance arises from both the voice prosthesis and the pharyngoesophageal segment. The combined resistance to airflow from the voice prosthesis and the pharyngoesophageal segment results in a 7.5 times greater phonatory airflow resistance in PWL compared with laryngeal speakers (Bohnenkamp et al. 2012). In sustained phonation, tracheoesophageal speakers therefore use expiratory breath flow less efficiently than laryngeal speakers.

Alaryngeal speech is associated with greater effort and fatigue levels when compared to laryngeal speech (Searl and Knollhoff 2018). This may arise from the way tracheoesophageal speakers compensate for inefficiencies in speech respiration and increased airway resistance. Tracheoesophageal speakers use more of their inspiratory reserve volume to fill the lungs prior to initiating speech and continue past their resting expiratory level, using more of their functional residual capacity (the volume of air remaining in the lungs after normal exhalation) during speech production (Bohnenkamp et al. 2011), which reduces phrase length and maximum phonation time (MPT) (Ward et al. 2007). This is exemplified by the finding that tracheoesophageal speakers use higher lung volumes for speech whilst producing fewer syllables per breath compared to laryngeal speakers (Bohnenkamp et al. 2011).

Whilst EMST may provide benefit to the impairments to respiration and cough, a significant challenge of the existing evidence base is that studies have been carried out on laryngeal speakers, whereas post‐laryngectomy anatomy is considerably different. Therefore, it is uncertain whether study outcomes are transferable to PWL. One pilot study has been completed on the use of EMST in laryngectomy (Van Sluis et al. 2020). This study found that EMST improved MEP and loudness. However, the primary focus of the study was feasibility, safety and compliance rather than cough and secretion burden or potential secondary impact on self‐reported outcomes and perceptual clinician‐rated assessment of voice quality. Therefore, this feasibility study aims to investigate these areas with the hypothesis that EMST may increase MEP after laryngectomy, which would improve cough function, secretion clearance and breath support. In turn, improvement in cough function, secretion clearance and breath support may improve perceptual features of tracheoesophageal voice and communication‐related quality of life.

### 1.1 Aims

1. To investigate the feasibility and acceptability of EMST after laryngectomy
2. To explore the impact of EMST on the following:
3. Respiratory function measured by peak flow (PF), forced expiratory volume in 1 s (FEV1) and MEP
4. Participant‐reported outcome measures (PROMs) of cough and secretion burden, voice‐related QOL and communication by using the Cough and Sputum Assessment Questionnaire (CASA‐Q) (Crawford et al. 2008), Voice‐Related Quality of Life (V‐RQOL) (Hogikyan and Sethuraman 1999) and the Self‐Evaluation of Communication Experiences after Laryngectomy (SECEL) (Blood 1993), respectively
5. Tracheoesophageal voice quality, using the SToPS (Hurren et al. 2019), MPT and maximum decibels on phonation (dB)

## 2 Materials and Methods

### 2.1 Study Design

This feasibility study was of a repeated measures design. As an early exploratory study, it did not include a control group. However, a double baseline and follow‐up data collection points were implemented as control aspects.

### 2.2 Ethical Approval and Consent

The study received ethical approval from the City St Georges, University of London Research Ethics Committee (reference no. ETH2223‐1307). All participants gave informed written consent to participate.

### 2.3 Recruitment and Participant Criteria

Participants were recruited via social media, patient and professional networks between 28 November 2022 and 20 January 2023. The recruitment target was 10–12 participants. It was acknowledged that the study was underpowered to evaluate the efficacy of EMST in PWL, but this was a justifiable limitation, given the exploratory nature and the primary aim of collecting feasibility and acceptability data. Furthermore, the small population size of PWL influenced the recruitment target.

The study recruited PWL who use a voice prosthesis to communicate. Participants were required to be cancer‐free and at least six months post‐surgery and/or adjuvant treatment to ensure sufficient healing. Participants were required to travel to the university on four occasions and to have sufficient dexterity to use an EMST device (or have carer support available to do this). The following exclusion criteria were set, according to the EMST150 device manufacturer's contraindications:

- Asthma
- Ruptured ear drum or other condition of the ear
- Pregnancy
- Uncontrolled gastroesophageal reflux
- Uncontrolled hypertension
- Abdominal hernia or recent abdominal surgery
- Tracheostomy tube in situ
- Acute stroke
- Complex cardiac issues
- History of pneumothorax
- Recent surgery to the upper body
- Lung transplant or recent resection
- Dental abscess or osteoradionecrosis
- Epistaxis or active haemoptysis

### 2.4 Setting

Participants attended assessments within a small sound‐treated room at the university. Weekly check‐in appointments were held in‐person or via virtual modalities according to participant preference.

### 2.5 Outcome Measures

Measures were collected in the domains of feasibility, acceptability, respiratory function, PROMs and tracheoesophageal voice quality.

Feasibility outcomes included the proportion of participants eligible from those who expressed interest in participating, the proportion of eligible participants who consented to take part, and the number of participants who completed the intervention.

Acceptability was assessed by reviewing participant adherence to protocol and their experience of the intervention via a practice diary. At follow‐up assessment, participants additionally completed a semi‐structured discussion with the research team. Based on existing research (Van Sluis et al. 2020), the risk of adverse events was low. Formal criteria for study cessation were not defined in advance; however, study progress was monitored, and no stoppage on safety grounds was required.

Respiratory function was assessed using PF, FEV1 and MEP. MEP is a measurement of the maximal strength of the respiratory muscles on exhalation (Obando et al. 2012), whereas PF and FEV1 are measures of expiratory airflow (Culver 2012, p133). PF measures the amount of air exhaled with effort, whilst FEV1 measures the amount of air that can be forcefully exhaled in 1 s. At each timepoint, participants completed three repetitions of maximum exhalation into a peak flow meter (for PF and FEV1) and a pressure manometer (for MEP) via the neck stoma. The mean average of the three trials was recorded as the outcome.

Few laryngectomy‐specific PROMS exist; therefore, measures were chosen due to a precedent for their use in existing laryngectomy research.  The Cough and Sputum Assessment Questionnaire UK English version (CASA‐Q), (Crawford et al. 2008) was used to measure cough and secretion burden across the following four domains: cough symptoms, cough impact, sputum symptoms and sputum impact. The CASA‐Q was developed and validated for chronic bronchitis or chronic obstructive pulmonary disease; however, it has been used in laryngectomy research (Longobardi et al. 2022; Ward et al. 2023). A lower score indicates greater severity.

The Voice‐Related Quality of Life questionnaire (V‐RQOL) (Hogikyan and Sethuraman 1999) assesses the impact of voice impairment in the domains of physical functioning and social‐emotional. A higher score indicates greater severity of impact. The V‐RQOL was developed and validated for laryngeal voice users (Hogikyan and Sethuraman 1999; however, it has been used widely in laryngectomy voice research (Grillo et al. 2021; Cocuzza et al. 2020; Agarwal et al. 2015; Kazi et al. 2006). Construct validity of the V‐RQOL in the laryngectomy population has been investigated, identifying that a modified scoring procedure may be required to increase validity in alaryngeal populations (Bornbaum et al. 2014). The above‐mentioned studies did not use the modified V‐RQOL scoring, however, therefore for comparability it was not used in this study.

The Self‐Evaluation of Communication Experiences after Laryngectomy (SECEL) (Blood 1993) is a self‐rating measure designed specifically for PWL, which has undergone validity and reliability testing. The SECEL consists of 35 items relating to the experience of communication after laryngectomy, which are separated into General, Environment and Attitudinal subscales. General items highlight how people experience conversational situations, whereas the Environmental domain indicates how communication is experienced in different environments; the Attitudinal domain describes attitudes towards speech, self‐perception and perception of others (Rodrigues et al. 2023).  Higher scores indicate greater communication difficulty.

Tracheoesophageal voice quality was measured using MPT and maximum dB on sustained vowel phonation, using the mean average of three trials. Voice recordings of The Rainbow Passage (Fairbanks 1960) were used for perceptual voice rating. Recordings were anonymised and randomised using an online random number generator prior to blinded perceptual assessment by an SLT experienced in laryngectomy rehabilitation, using the SToPS (Hurren et al. 2019). The SToPs is a clinician‐rated 14‐item scale, which assesses perceptual parameters of tracheoesophageal voice. The SToPS has established validity, intra‐rater reliability (Hurren et al. 2019) and inter‐rater reliability for the majority of items (Coffey et al. 2019); and is the only scale designed to perceptually rate tracheoesophageal voice. However, minimally important clinical differences and sensitivity to change are not yet established.

### 2.6 Equipment

**FIGURE 1:**
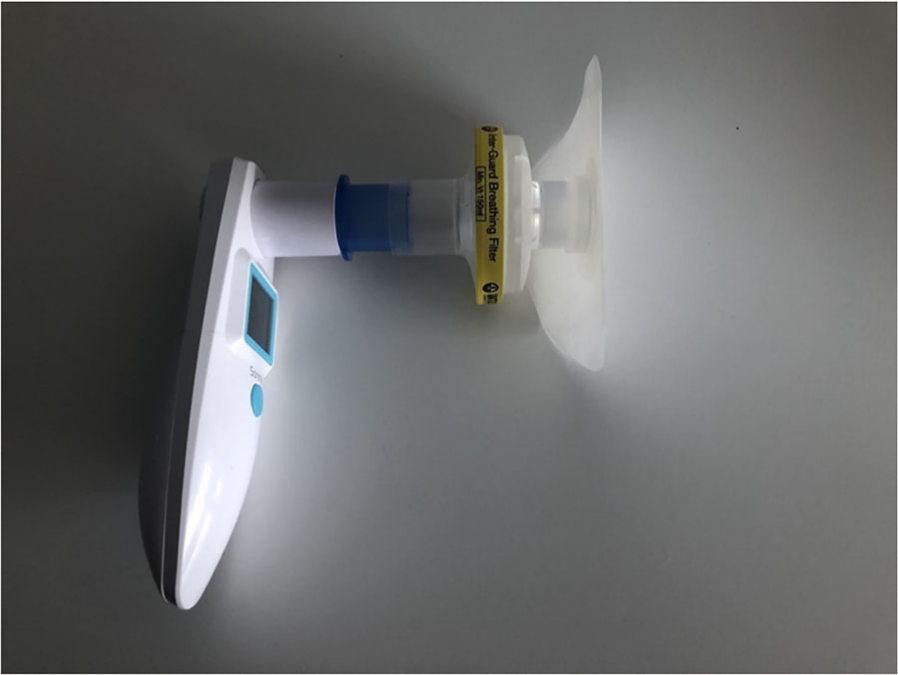
Attachment of PF/FEV1 meter to baseplate.

An EMST150 device (Aspire Respiratory Products) was used. The device was connected to a baseplate via the round mouthpiece for use at the neck stoma. Voice recordings were taken using a Shure SM137 condenser microphone at a 30‐cm microphone‐to‐mouth distance, connected to a Zoom H6 digital audio recorder. A Sauter SU130 sound level meter was sited directly under the microphone. A Sonmol peak flow meter was used to measure PF and FEV1 via an Intersurgical Inter‐guard breathing filter fitted to the mouthpiece of the EMST device and placed into a baseplate (Figure 1). An In‐Health pressure manometer was used to measure MEP with a manometer adaptor kit placed into the participant's normal baseplate.

### 2.7 Protocol

Participants attended two baseline assessments with a two‐week interval between them (*t*1 and *t*2), a post‐intervention assessment five weeks later (*t*3) and a follow‐up assessment after a six‐week maintenance period (*t*4).

Between *t*2 and *t*3, participants completed the EMST intervention. At the *t*2 baseline visit, participants were shown how to use the EMST device and instructed on the protocol for self‐delivery of the 5‐week EMST intervention. The device was set at the starting value of 75% of the participant's MEP. During the intervention period, participants were contacted weekly by a researcher to provide advice if required, to discuss weekly adherence and to gain qualitative feedback. Dependent on participant preference, weekly contact occurred via a combination of video call, virtual meeting, email, text chat or face‐to‐face visit.

At *t*3, participants attended for post‐intervention assessment and instruction on dosage for the six‐week maintenance period. After the maintenance period, participants attended the final assessment (*t*4). During the maintenance period, participants did not have routine weekly check‐ins; however, they were able to contact the team at any point if required.

### 2.8 Intervention Description

To complete the five ‐week EMST intervention, participants were required to exhale forcefully into the hand‐held device 25 times per day. This was completed in sets of five repetitions, five times per day, for five days per week, comprising a total dosage of 625 breaths over the five‐week intervention. Participants increased the pressure threshold of the device periodically to sustain work at 75% of MEP as they became accustomed to the expiratory pressure required. Participants were not advised to plug the voice prosthesis during practice.

During the maintenance period, participants followed a reduced dosage of 25 breaths per day, for two days per week, for six weeks. This comprised a total maintenance dosage of 300 breaths. Participants continued to adjust the device as required to work at 75% of MEP. Supporting material 1 describes the intervention using the TIDieR checklist (Hoffmann et al. 2014).

### 2.9 Data Analysis

Descriptive statistics reported participant characteristics, feasibility and quantitative acceptability outcomes and scores on outcome measures. Qualitative data on acceptability obtained from practice diaries and participant reports were analysed using content analysis. PROMs were scored according to instructions prior to statistical analysis. For dB, MPT, PF, FEV1 and MEP values, the mean average of three trials was calculated prior to statistical analysis.

The SToPs does not provide an overall score for analysis; therefore, individual items were inspected descriptively, as intended, and a composite score was calculated for inferential statistical analysis. To calculate the composite score, 11/14 of the SToPs’ individual items were summed. Accent and Reading scores were excluded from the composite score as these items serve to identify potential bias in the rating clinician and are not expected to change over time. Tonicity was excluded for the following two reasons: (a) it was not found to be as reliable as other parameters (Coffey et al. 2019); (b) tonicity is rated via an 11‐point bipolar scale that was incompatible with the scoring of the other items, as both high and low scores indicate impairment. Prior to inclusion in the composite score, the Positive Features item was reverse scored to be consistent with other SToPs items, where higher scores indicate a greater level of impairment.

Statistical analysis on outcome measures scores across time was carried out with SPSS using one‐way repeated measures ANOVA or Friedman test to explore the pattern of change. Given the exploratory nature of this study, adjustments for multiple comparisons were not used, but *p* values are given together with effect sizes. Effect sizes were calculated by partial eta squared using interpretation values from Statology (Zach 2021), or Kendall's *W* for non‐parametric data, using interpretation values from Landis and Koch (1977).

## 3 Results

### 3.1 Participant Characteristics

Ten PWL took part in the study. Participants were predominantly male and White‐British with a mean age of 62.2 years (SD: 14.94). All participants had total laryngectomy, with 40% of participants having total laryngectomy with pectoralis major flap. The median time post‐surgery was 29 months (IQR: 17.25–133.00), and 70% of participants had received radiotherapy before or after surgery.  Table 1 details participant characteristics.

**TABLE 1:** Participant characteristics (*n* = 10).

Participant characteristics	Mean (SD) and range
Sex	Male: 8
	Female: 2
Age	Mean: 62.2 years, SD: 14.94
	Range: 37–80 years
Ethnicity	White British: 8
	Black‐Caribbean: 1
	White European: 1
Employment status	Retired: 7
	Part‐time work: 1
	Full‐time work: 2
Months post‐surgery	Mean: 61.1 months, SD: 60.99
	Median: 29 months, IQR: 17.25–133.00
	Range: 6–172 months
Type of surgery	Total laryngectomy: 6
	Total laryngectomy with pectoralis major flap: 4
Radiotherapy	None: 3
	Pre‐surgery: 4
	Post‐surgery: 3
Type of prosthesis	Blom‐Singer Classic indwelling prosthesis: 5
	Blom‐Singer Increased Resistance indwelling prosthesis: 1
	Blom‐Singer Low‐Pressure ex‐dwelling^a^ prosthesis: 1
	Provox Vega indwelling prosthesis: 1
	Provox NID non‐indwelling prosthesis: 2

^a^ Ex‐dwelling prostheses may also be termed ‘non‐indwelling’ and require a tab to be maintained at the peri‐stomal area when the prosthesis is in situ.

### 3.2 Feasibility

During the recruitment period, 14 PWL expressed an interest in participating. Of the 14, two people were ineligible to take part due to contraindications (poorly controlled reflux, *n* = 1; less than 6 months post‐surgery, *n* = 1). Of the 12 eligible PWL, 10 consented to take part in the study, with two PWL declining due to the requirement to travel to the four in‐person assessments. Both lived at a significant distance from the study location. The ten participants who consented all completed the intervention and attended all assessments.

### 3.3 Acceptability

During the intervention period, all participants completed the set dosage of 625 breaths. However, adherence to the prescribed frequency of 25 breaths per day for five days per week varied in two participants. One participant completed the dosage over seven weeks due to a mild Covid‐19 infection, which impaired respiratory function for several days. Another participant completed the dosage in six weeks due to an unrelated rib injury, which required cessation of practice for one week.

During the maintenance period, 80% of participants completed the set dosage of 300 breaths, however, one participant varied the frequency each week to facilitate the practice around daily commitments. Two participants did not complete the full maintenance dosage, due to uncertainty of the benefit of additional practice (*n* = 1) and difficulty sustaining additional practice around other commitments (*n* = 1). These participants completed 33% and 83% of the maintenance dosage, respectively.

Participants reported that the EMST protocol was acceptable and could be accommodated into their daily routine after a short period of establishing the routine. Two participants considered their baseplate usage within the timing of practice, noting that after several hours of wear, the baseplate seal was less resistant to the pressure of the expiratory breath. Practice was therefore timed to accord with more recent baseplate placement; however, for one participant, this resulted in reduced lifespan of baseplate seal after practice.

Nine of the ten participants felt EMST practice was beneficial. Participants described increased exercise tolerance on walking or stair climbing, the sensation of greater lung expansion on inhalation and ease of mucus clearance, with changes typically reported after 2 weeks of practice. One participant observed easier mucus clearance with lowered nebuliser use, yet the pattern of mucus clearance altered, resulting in increased expectoration during the day, which was inconvenient. Two participants queried whether EMST may benefit swallow function, due to the sensation of pharyngeal constrictor engagement during exhalation into the device. This was not investigated during the study, however.  All participants expressed satisfaction with the frequency of contact with the research team and the offer of multi‐modal weekly appointments.

#### 3.3.1 Equipment Issues and Problem Solving

Participants were advised to use the nose clips or pinch the nose when practising, if they experienced egressive airflow via the prosthesis to the nose on exhalation into the device. This was reported by one participant who pinched the nose to improve the air seal. Two participants reached the maximum pressure of the device during the five‐week intervention period.

All participants used the device independently; however, device adaptations were necessary to enable safe and efficient use at the neck stoma. Most participants completed practice with their usual baseplate, whilst three participants found that the seal was insufficient to prevent air leak between the baseplate and the skin during forceful exhalation. These participants changed to the use of a strongly adhesive baseplate during practice to improve the seal.

**FIGURE 2:**
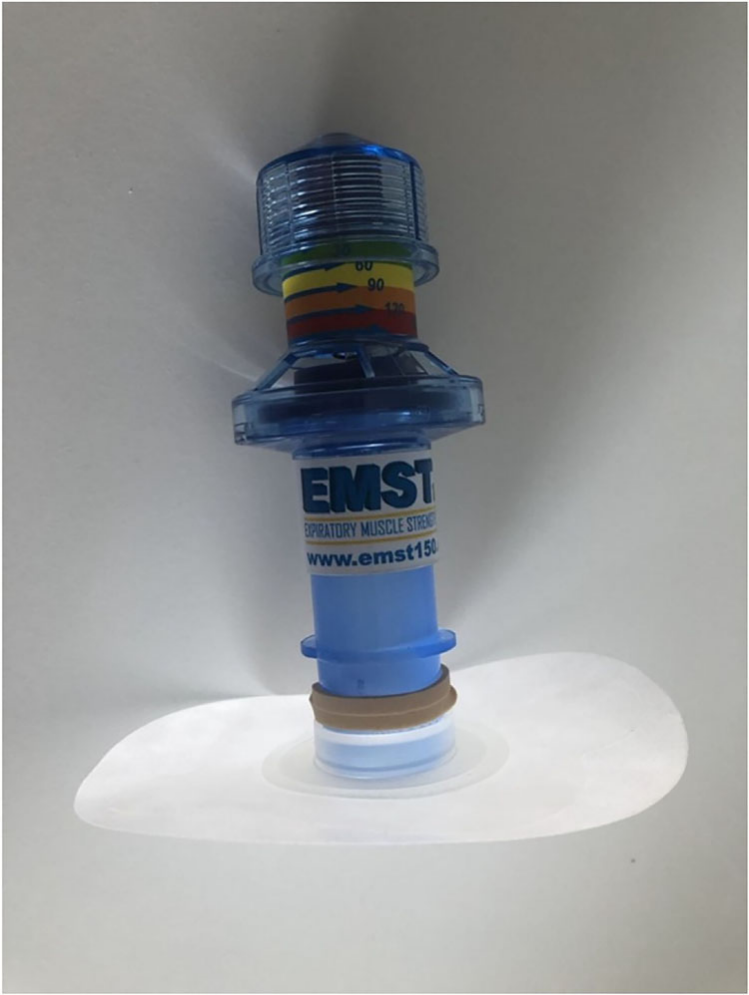
Placement of elastic bands on device to improve seal.

Participants were instructed to place the device into their baseplate using the round mouthpiece connector, which has a 22‐mm diameter. Depending on brand, typical baseplate diameters are 22–23 mm. Air leak between the baseplate and the device was experienced by most participants but exacerbated for those using 23‐mm diameter baseplates. This issue was resolved by using elastic bands wrapped around the device mouthpiece, which enabled a complete seal between the device and the baseplate (Figure 2). In addition, the placement of elastic bands prevented participants from inserting the device too far into the baseplate, which could cause peristomal skin irritation.

**FIGURE 3:**
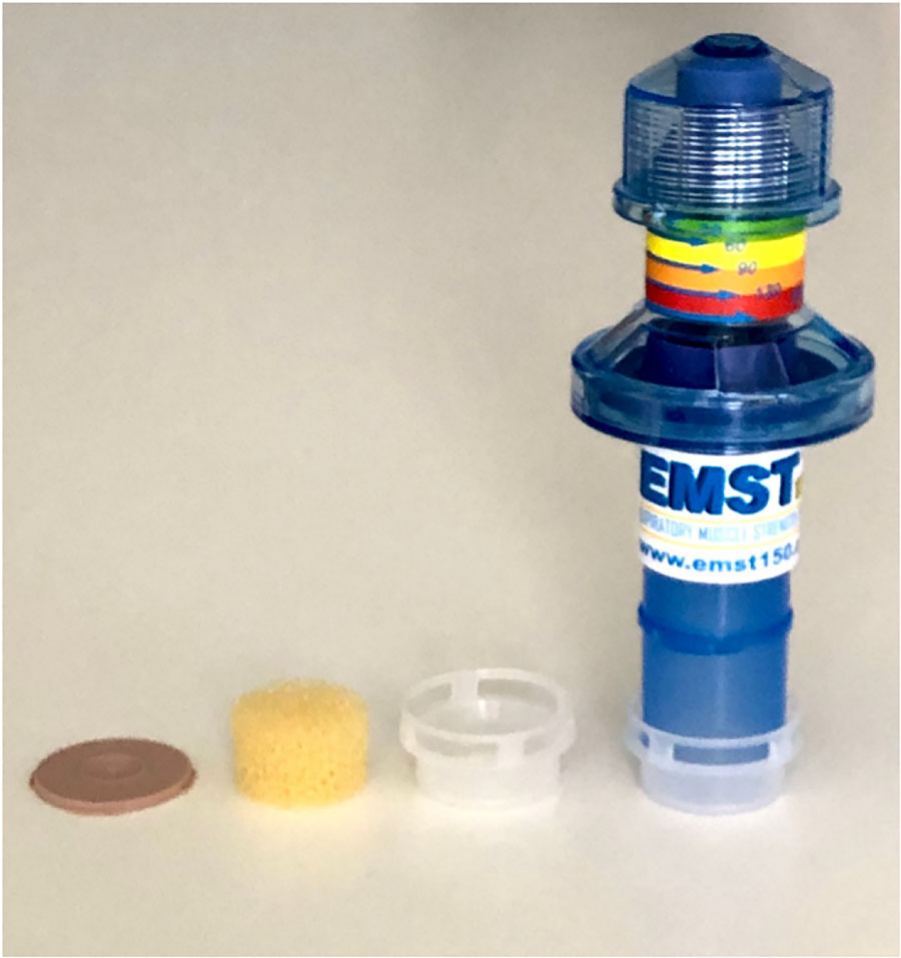
Deconstructed HME casing for device.

Participants were encouraged to experiment with the device to explore facilitatory solutions beyond the use of elastic bands. One participant used a deconstructed HME as a casing for the device mouthpiece, which was an alternative way to improve device seal with the baseplate (Figure 3). A second participant adapted the In‐Health manometer adapter to use as an alternative casing for the device mouthpiece, as discussed in the following section.

#### 3.3.2 Safety Considerations

**FIGURE 4:**
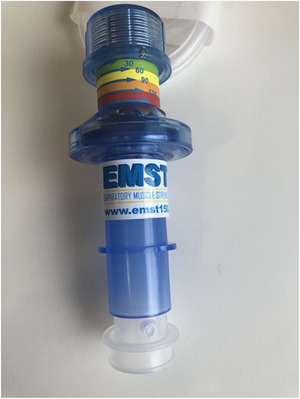
Device with modified In‐Health manometer adapter.

**FIGURE 5:**
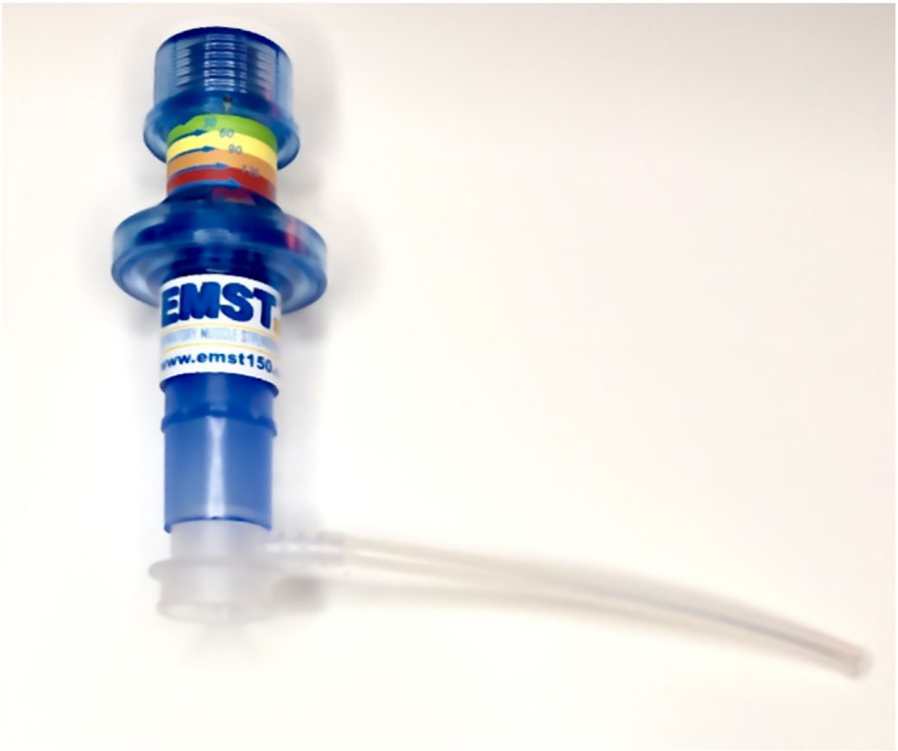
Device with In‐Health manometer adapter tubing.

It is not possible to inhale through the device when in situ; therefore, it is essential to remove the device promptly from the neck stoma after exhalation in order to inhale. This presents a significant potential airway safety issue for PWL that use EMST. Clinicians must ensure that they have comprehensively assessed the individual PWL's ability to consistently remove the device from the neck stoma to ensure that there is no risk of airway obstruction from the device. Two participants reported reduced hand dexterity that caused a small delay in removing the device rapidly after exhalation; therefore, a participant‐led solution was explored to increase safety and comfort of use. An In‐Health manometer adapter was modified to create a connector between the device and the baseplate, with a hole cut into the adapter to allow airflow (Figure 4). Participants were then able to cover the hole with a finger during exhalation into the device, and to remove the finger for inhalation with the device in situ. This technique was preferred by one participant for efficiency, rather than a dexterity issue. Where participants presented with a deep, recessed stoma, the manometer tubing could be left attached to enable inhalation through the tubing (Figure 5). It is of note that these solutions require an unorthodox modification and usage of manometry equipment, however.

#### 3.3.3 Adverse Events

Two participants reported minor peristomal skin irritation. The causal factor was found to be the use of excessive contact pressure to prevent air leak when connecting the device at the neck stoma. Participants were given advice on technique, and the use of elastic bands as stated above was implemented. This improved air seal and reduced the contact pressure required on placing the device. The skin irritation resolved within a few days of review.  One participant reported light‐headedness in the first week of practice after completing all daily breaths in one set. This resolved immediately after the participant was reminded to complete breaths in smaller sets. No participants reported dislodgement of the voice prosthesis during the study.

### 3.4 Outcome Measures

Table 2 presents group outcome measure results across timepoints. Where data were non‐parametric, median scores and interquartile ranges are additionally reported in italics. 

**TABLE 2:** Group outcome measure results across time and statistical comparisons.

Outcome measure	Baseline 1	Baseline 2	Post‐intervention	Follow‐up	Repeated measures
	Mean (SD)	Mean (SD)	Mean (SD)	Mean (SD)	
	Median (IQR)	Median (IQR)	Median (IQR)	Median (IQR)	ANOVA or Friedmans
Respiratory					
FEV1 (L/s)	1.12 (0.32)	0.94 (0.27)	1.35 (0.28)	1.40 (0.31)	*p* < 0.001
Peak flow (L/min)	220.93 (64.72)	215.73 (76.13)	266.40 (67.46)	270.60 (64.09)	*p* = 0.002
				*250.84 (236.83–293.59)*	
Maximum expiratory pressure (cmH_2_O)	119.40 (21.79)	120.40 (23.76)	136.03 (14.97)	134.37 (14.33)	*p* = 0.004
Participant‐reported measures					
CASA‐Q COUS	57.50 (13.86)	63.33 (16.76)	63.33 (24.60)	60.83 (17.59)	*p* = 0.45
	*62.50 (54.17–66.67)*		*66.67 (58.33–77.08)*		
CASA‐Q COUI	74.07 (17.86)	70.32 (18.82)	80.32 (22.78)	76.25 (18.23)	*p* = 0.04
	*75.00 (64.85–85.16)*	*75.00 (57.82–82.81)*	*84.38 (77.35–92.97)*	*79.70 (67.19–88.28)*	
CASA‐Q SPUS	49.17 (20.20)	53.75 (20.07)	55.00 (27.83)	59.17 (23.72)	*p* = 0.20
	*54.17 (41.67–66.67)*				
CASA‐Q SPUI	60.41 (19.46)	59.17 (22.97)	65.83 (25.29)	64.58 (24.71)	*p* = 0.05
		*70.83 (39.58–75.00)*	*75.00 (58.33–83.33)*		
V‐RQOL	26.20 (10.52)	26.00 (11.94)	24.40 (10.55)	24.10 (12.73)	*p* = 0.36
	*21.00 (17.75–36.00)*	*21.00 (17.75–36.25)*	*20.00 (16.75–30.75)*	*18.00 (14.75–36.75)*	
SECEL total score	39.00 (21.88)	38.80 (25.75)	33.40 (21.22)	33.60 (28.00)	*p* = 0.05
	*29.00 (22.00–61.25)*	*26.50 (18.75–65.50)*		*22.00 (12.75–53.25)*	
SECEL General score	5.70 (3.43)	5.20 (4.16)	4.90 (3.41)	4.60 (3.69)	*p* = 0.44
SECEL Environmental score	20.20 (8.47)	19.20 (10.38)	16.90 (7.77)	17.10 (11.39)	*p* = 0.01
		*15.00 (11.00–29.25)*		*12.50 (8.75–25.00)*	
SECEL Attitudinal score	13.10 (11.19)	14.20 (11.85)	11.60 (10.86)	11.90 (13.50)	*p* = 0.046
	*7.50 (6.00–23.00)*	*7.50 (6.75–26.00)*	*6.00 (3.75–23.00)*	*6.00 (3.00–21.50)*	
Objective voice measures					
Maximum phonation time (s)	10.59 (5.26)	10.46 (9.44)	9.44 (6.79)	8.97 (6.58)	*p* = 0.52
			*8.01 (3.97–14.18)*	*7.30 (3.96–13.20)*	
Decibels	74.88 (6.45)	76.15 (5.54)	79.56 (5.72)	82.05 (4.91)	*p* ≤ 0.001
SToPS Perceptual scale					
Composite score	13.30 (8.42)	13.10 (7.71)	12.60 (8.75)	13.90 (9.94)	*p* = 0.69
Overall voice quality	1.60 (0.97)	1.60 (0.97)	1.50 (0.85)	1.70 (0.95)	*p* = 0.27
Strain	1.40 (0.97)	1.30 (1.16)	1.10 (0.99)	1.40 (1.35)	*p* = 0.54
Wetness	0.70 (1.06)	0.70 (0.95)	0.70 (0.95)	0.90 (1.20)	*p* = 0.87
	*0.00 (0.00–1.25)*	*0.50 (0.00–1.00)*	*0.50 (0.00–1.00)*	*0.50 (0.00–1.50)*	
Volume	1.10 (1.10)	1.10 (1.10)	1.10 (1.20)	1.40 (1.07)	*p* = 0.33
Social acceptability	1.80 (1.03)	1.80 (0.92)	1.70 (0.95)	1.70 (1.06)	*p* = 0.77
Whisper	1.30 (0.95)	1.50 (0.85)	1.40 (1.07)	1.40 (1.07)	*p* = 0.66
Intelligibility	0.80 (1.13)	0.70 (1.06)	0.90 (1.20)	0.80 (1.23)	*p* = 0.59
	*0.00 (0.00–2.00)*	*0.00 (0.00–1.25)*	*0.50 (0.00–1.50)*	*0.00 (0.00–1.50)*	
Stoma	1.10 (0.87)	0.80 (0.63)	0.60 (0.52)	0.80 (1.03)	*p* = 0.45
				*1.00 (0.00–1.25)*	
Fluency	0.70 (0.82)	0.70 (1.16)	0.70 (1.06)	1.0 (1.05)	*p* = 0.23
		*0.00 (0.00–2.00)*	*0.00 (0.00–1.25)*		
Articulation	0.80 (1.14)	0.80 (1.03)	0.70 (0.95)	0.80 (1.14)	*p* = 0.85
	*0.00 (0.00–2.00)*	*0.50 (0.00–1.25)*	*0.50 (0.00–1.00)*	*0.00 (0.00–2.00)*	
Positive features	2.00 (1.05)	2.10 (0.88)	2.20 (0.92)	2.00 (0.94)	*p* = 0.76
		*2.00 (2.00–3.00)*	*2.00 (2.00–3.00)*		
Tonicity	−0.90 (2.51)	−0.90 (2.77)	−1.00 (2.49)	−0.80 (2.78)	0.70
Accent	0.90 (0.99)	0.90 (0.99)	0.80 (1.03)	0.90 (0.99)	*p* = 0.73
	*1.00 (0.00–1.25)*	*1.00 (0.00–1.25)*	*0.50 (0.00–1.25)*	*1.00 (0.00–1.25)*	
Reading	0.40 (0.70)	0.20 (0.42)	0.20 (0.63)	0.10 (0.32)	*p* = 0.22
	*0.00 (0.00–1.00)*	*0.00 (0.00–0.25)*	*0.00 (0.00–0.00)*	*0.00 (0.00–0.00)*	

#### 3.4.1 Respiratory Function

There was a significant improvement in FEV1 values post‐intervention *F*(3, 27) = 13.10; *p* <0.001, with a large effect size (*η*_p_^2^ = 0.59). There was no significant difference between baseline scores (*p* = 0.21), whereas there was a significant increase in FEV1 between Baseline 2 and post‐intervention and follow‐up (*p* = 0.002; *p* = 0.01). There was no significant decrease in score between post‐intervention and follow‐up (*p* = 1.00).

MEP values improved post‐intervention *F*(3, 27) = 5.71; *p* = 0.004, with a large effect size (*η*_p_^2^ = 0.39). There was no significant difference in baseline mean scores (*p* = 1.00), which were 119.40 cmH_2_O (21.79) at Baseline 1 and 120.40 cmH_2_O (23.76) at Baseline 2. Mean MEP score increased significantly between Baseline 2 and post‐intervention to 136.03 cmH_2_O (14.97); (*p* = 0.047). Whilst there was no significant difference in MEP between post‐intervention and follow‐up (*p* = 1.00), the mean score decreased slightly at follow‐up to 134.37 cmH_2_O (14.33), which was not significantly different from Baselines 1 and 2 mean scores (*p* = 0.23, *p* = 0.33, respectively).

Peak flow improved from baseline to post‐intervention and follow‐up, Friedman χ^2^(3) = 14.52, *p* = 0.002 with a moderate effect size (*W* = 0.484). Median (IQR) scores were 224.00 L/min (187.75–245.50) at Baseline 1, 213.67 L/min (146.17–266.33) at Baseline 2, increasing to 253.00 L/min (214.75–342.99) post‐intervention and 250.84 L/min (236.83–293.59) at follow‐up. Post hoc comparisons demonstrated no significant differences between Baselines 1 and 2 (*p* = 0.58), or between post‐intervention and follow‐up (*p* = 0.39), but significant increases between the two baseline scores and post‐intervention and follow‐up scores (*p* = 0.03–0.01)

#### 3.4.2 Participant Reported Outcome Measures

Median V‐RQOL scores showed a downward trend; however, no change across timepoints was significant, Friedman χ^2^(3) = 3.21, *p* = 0.36, with a small effect size (*W* = 0.11). Median (IQR) scores were consistent between Baselines 1 [21 (17.75–36.00)] and 2 [21 (17.75–36.25)], followed by slightly lower scores post‐intervention [20 (16.75–30.75)] and at follow‐up [18 (14.75–36.75)].

On the CASA‐Q, COUS scores did not change significantly following EMST, Friedman 𝜒 ^2^(3) = 2.67, *p* = 0.45, with a small effect size (*W* = 0.09). Additionally, no significant change in scores was observed for SPUI (Friedman 𝜒^2^(3) = 7.71, *p* = 0.052) with a small effect size (*W* = 0.26) or SPUS (Friedman 𝜒^2^(3) = 4.64, *p* = 0.20) with a small effect size (*W* = 0.15). However, SPUS scores did meet the minimal clinically important difference level for reduction in sputum symptoms between Baseline 1 [median (IQR) = 54.17 (41.67–66.67] and follow‐up [median (IQR) = 66.65 (33.33–77.08)]. COUI scores were significantly improved post‐intervention (Friedman 𝜒^2^(3) = 7.86, *p* = 0.049), with a small effect size (*W* = 0.26), demonstrating a reduction in cough impact between median scores at Baseline 2 [median (IQR) = 75.00 (57.82–82.81)] and post‐intervention [median (IQR) = 84.38 (77.35–92.97)], *p* = 0.02. Post‐intervention improvement was sustained at follow‐up with no significant difference in scores, *p* = 0.23.

On the SECEL, there were no significant differences in the total score (Friedman 𝜒^2^(3) = 7.61, *p* = 0.05 with a small effect size, *W* = 0.25) or the general domain score (*F*(3, 27) = 0.93, *p* = 0.44 with a medium effect size, *η*_p_^2^ = 0.09) across timepoints. In the environmental domain, participants’ median scores improved over time (Friedman 𝜒 ^2^(3) = 11.40, *p* = 0.01 with a small effect size, *W* = 0.38). Environmental domain median (IQR) scores were significantly lower at post‐intervention assessment [14.50 (11.25–25.50)] compared with Baseline 1 [17.00 (14.75–28.50)], *p* = 0.008, and Baseline 2 [15.00 (11.00–29.25)], *p* = 0.04. There were no significant differences in baseline median scores (*p* = 0.17) or between post‐intervention and follow‐up (*p* = 0.83); however, median (IQR) follow‐up scores [12.50 (8.75–25.00)] were not significantly different to Baselines 1 (*p* = 0.08) and 2 (*p* = 0.13). Attitudinal domain scores also improved over time (Friedman 𝜒^2^(3) = 8.01, *p* = 0.046 with a small effect size, *W* = 0.27). Post hoc comparisons demonstrated significantly lower median (IQR) scores post‐intervention [6.00 (3.75–23.00)] compared with Baseline 2 [7.50 (6.75–26.00)]; *p* = 0.02. There was no significant difference in median scores between Baselines 1 [7.50 (6.00–23.00)] and 2 (*p* = 0.08); or post‐intervention to follow‐up score [6.00 (3.00–21.50)]; *p* = 0.89.

#### 3.4.3 Measures of Voice Quality

Mean maximum dB were not significantly different (*p* = 1.00) between baseline values of 74.88 (6.45) at Baseline 1 and 76.15 (5.54) at Baseline 2. Participants did increase mean maximum decibels post‐intervention, *F*(2.05, 18.46) = 12.47; *p* < 0.001, with a large effect size (𝜂_p_^2^ = 0.58). Post hoc comparison indicated that participants increased their scores between Baseline 1 and follow‐up (82.05, SD: 4.91), *p* = 0.013; and between Baseline 2 and post‐intervention (79.56, SD: 5.72), *p* = 0.001 and follow‐up, *p* = 0.015. Decibel increase was sustained during the maintenance period with no significant differences between post‐intervention and follow‐up scores (*p* = 0.67).

No significant changes were found in MPT in seconds across timepoints, Friedman 𝜒2(3) = 2.28, *p* = 0.52, with a small effect size (*W* = 0.08). Median (IQR) values showed a trend of attenuating MPT post‐intervention and at follow‐up: Baseline 1 [11.29 (5.28–15.38)], Baseline 2 [11.65 (4.35–16.07)], post‐intervention [8.01 (3.97–14.18)], follow‐up [7.30 (3.96–13.20)], however, the reduction in MPT was not significant.

There were no significant differences in the composite score, *F*(3, 27) = 0.50; *p* = 0.69, overall voice quality *F*(3, 27) = 1.39; *p* = 0.27 or individual item scores of the SToPs. Visual inspection of the data did not demonstrate any clear pattern of change. This indicated no change in perceptual voice quality between baselines or post‐intervention, as assessed by an experienced clinician.

## 4 Discussion

This study investigated the feasibility and preliminary efficacy of EMST after total laryngectomy. The study found that EMST is feasible for PWL and was indicative of potential benefit in respiratory measures and voice loudness in decibels; clinician‐rated voice quality was unchanged, however.  Participants additionally reported reduced impact of cough, improved attitude towards speech and of communicating in different environments as measured by the CASA‐Q and SECEL. However, as a small feasibility study, with a self‐selecting group of motivated participants, outcomes should be interpreted tentatively.

Ten participants completed the intervention with high adherence to the protocol, demonstrating that EMST is feasible and acceptable after laryngectomy. Two additional eligible participants did not consent to take part in the study due to long travel distances.

All participants required device adaptations to prevent air leak and, in some cases, to support insertion of the device at an appropriate depth. Simple adaptations were effective with the use of low‐cost materials or modification to commonly used tracheostoma equipment. It is recommended that a highly adhesive baseplate is used during EMST practice, with associated peristomal skincare advice.

Safety solutions were required if the device could not be removed promptly from the neck stoma to allow for inhalation. It is essential that clinicians routinely assess the individual's ability to consistently remove the device from the neck stoma to enable prompt inhalation, including assessment of hand and arm dexterity. This ability must be assessed routinely for all PWL using EMST as standard practice to reduce risk.  Shoulder mobility may be impaired after laryngectomy (Rauchenwald et al. 2021; Yeh et al. 2017), impacting the ability to raise the arm towards the neck stoma; therefore, this may present a risk in removing the device for people with shoulder dysfunction or other impairment to dexterity. Studies on the use of EMST or device‐based inspiratory muscle training via tracheostomy are infrequent and do not report on device removal or air leak (Clayton et al. 2022; Elkins and Dentice 2015; Santos‐Pascotini et al. 2014; Martin et al. 2011). However, since these studies took place in critical care units, it may be expected that healthcare professionals were present to assist with device insertion and removal. Furthermore, equipment is designed to attach more readily to a tracheostomy tube in contrast with laryngectomy products.

One prior pilot study of EMST use in laryngectomy exists (Van Sluis et al. 2020). Within the Van Sluis et al. (2020) study, an adapter was specially designed and produced to connect the device to the neck stoma. The adapter included an opening that the participant can use to inhale and occlude manually. To increase clinical transferability of the present study, a bespoke device was not developed during this study, as this would not be readily available for clinical usage. Low‐cost, accessible device modifications were therefore used; however, it is recommended that EMST device manufacturers develop adapters specifically to enable safe usage of the device within the laryngectomy population.

In this prior existing study, Van Sluis et al. (2020) also advocated for future testing without plugging of the voice prosthesis to ascertain whether improvement can still be gained. In the present study, participants were advised not to plug the prosthesis, with only one participant requiring nose‐pinch to reduce airflow via the prosthesis. As participants demonstrated significant gains in respiratory parameters without plugging the prosthesis, this suggests that plugging is not necessary to derive benefit from EMST.

Across all outcome measures, there were no differences between baseline assessments, indicating that participants’ respiratory function was stable prior to commencing EMST. Normative respiratory values have not yet been established for PWL. In comparison with normative values for the laryngeal population, the (predominantly male) participants’ average MEP at all timepoints was below the male lower threshold of 140 cmH_2_O (Ponce, Sankari and Sharma, 2024). Similarly, PF values at all timepoints were below the female and male lower threshold ranges of normal by mean age, using the EN13826 scale (Clement Clarke International 2004).

Study outcomes demonstrated a significant increase post‐intervention in MEP, albeit with a detraining effect at follow‐up. The increased MEP value is consistent with existing EMST research in the laryngeal population and with Van Sluis et al. (2020) relating to the laryngectomy population. In contrast with Van Sluis et al. (2020), measures of PF and FEV1 also increased over time, and gains were maintained at follow‐up. Increased PF with EMST was also observed in studies of elderly populations (Park et al. 2017) and after partial laryngectomy (Palmer et al. 2019). The differences in PF and FEV1 outcomes between this study and Van Sluis et al. (2020) may be explained by differences in participant characteristics, as mean baseline values for PF, FEV1 and MEP were lower than baseline values in Van Sluis et al. (2020). Van Sluis et al. (2020) described all their participants as ‘relatively fit’ which may have led to a ceiling effect. A measure of performance status or exercise tolerance was not included in this study to quantify this difference, however. Gains in respiratory function post‐intervention are consistent with qualitative participant reports of increased exercise tolerance in walking and stair climbing, and greater lung expansion on breathing. This finding has been reported in healthy subjects who reported reduced sensation of respiratory effort during treadmill‐based exercise, following EMST (Suzuki et al. 1995).

Two participants reached the maximum pressure threshold of the device before the end of the five‐week intervention, which may have curtailed their potential for further increases in respiratory outcome measures. This was also reported in Van Sluis et al. (2020). Future research into EMST post‐laryngectomy may benefit from the use of higher‐pressure threshold devices and exploration of this finding, such as the impact of altered anatomy and lack of laryngeal valving on expiratory force.

On PROMS, no significant change was found in the V‐RQOL. This scale has not been developed for the laryngectomy population, however and therefore may not be sensitive to change in subjective tracheoesophageal voice‐related differences. Outcomes of the SECEL demonstrated reduced impairment in the Environmental and Attitudinal domains. Change in Environmental score expresses how participants experience their voice in different environments, whereas change in Attitudinal score expresses how participants perceive their voice and how they are perceived by others (Johansson et al. 2008). Loudness is a frequent concern for tracheoesophageal speakers, with difficulty being understood in noisier environments. The improved Environmental and Attitudinal scores could therefore be interpreted as consistent with the increased maximum dB post‐intervention.

Participants reported easier mucus clearance following EMST, which correlated with a reduction in cough impact and severity of sputum as measured by the CASA‐Q. In the COPD population, who experience hypersecretion of mucus, improvement in CASA‐Q scores has been shown to relate closely to improved QOL (Shen et al. 2018). Amongst symptoms and concerns most closely relating to the CASA‐Q cough impact domain, people with COPD reported impact on social interactions and discomfort in talking to others. Whereas the sputum severity domain was related to difficulty and exhaustion arising from mucus clearance (Patalano et al. 2022). This may provide some rationale for the consistency in improved CASA‐Q and SECEL Attitudinal and Environmental scores produced by PWL in this study.

A significant increase in maximum dB was observed post‐intervention and maintained at follow‐up. This was consistent with Van Sluis et al. (2020). In a study of laryngeal speakers, expiratory muscle tension was increased when speaking louder (Huber et al. 2005); therefore, gains in MEP may have contributed to the increase in maximum dB found within this study. Conversely, there was a small, non‐significant decline in MPT across timepoints. Normative values for MPT are not established for PWL; however, a previous study which measured MPT in PWL reported a mean MPT of 7.0 s (Takeshita‐Monaretti et al. 2014), which was shorter than the findings of this study.  Increase in dB negatively influences MPT in laryngeal populations (Schmidt et al. 1988). This may suggest that as participants improved vocal intensity, breath support was modified, leading to slightly reduced MPT; however, the decrease across timepoints was not significant.

No changes were found in perceptual voice quality after EMST intervention. Participant scores on the items of the SToPs scale were within the range of no impairment to mild impairment at baseline, with low impact of reading or accent features. Similarly, mean tonicity was within the range of mildly hypotonic to normal. Therefore, there is potentially little room for improvement. However, it is worth noting that the clinician ratings of no‐to‐mild impairment were inconsistent with PROMs; and at all timepoints, participant‐reported scores met the threshold values for rehabilitation (Tuomi et al. 2015) in all SECEL domains.

The inconsistency of SToPs rating between participants and clinicians has been found in previous research involving auditory‐perceptual assessment of tracheoesophageal voice (Coffey et al. 2018). This may reflect a limitation of the SToPs as a clinician‐rated measurement tool; however, it is of note that inconsistency between service user and clinician rating is also reported in laryngeal voice research (Karnell et al. 2007; Kim and Choi 2009) and so points to a wider question on factors influencing service user and clinician rating of voice. Development and testing of perceptual tracheoesophageal voice outcome measures is required to establish sensitivity to change, clinically important values and correlation with participant experience of communication.

This study adds to the evidence base on post‐laryngectomy rehabilitation, demonstrating the feasibility and acceptability of EMST and improvements in some outcomes. Results should be interpreted with caution, however, due to the small sample size. Further testing is required to explore the efficacy of EMST in an appropriately powered study. A further limitation is the absence of normative respiratory, acoustic and perceptual outcomes for tracheoesophageal speakers, which limit interpretation of outcomes in the context of the wider laryngectomy population. After laryngectomy, anatomical changes result in decreased dead space of the respiratory tract; however, the impact of this on respiratory function is not yet established in literature (Ackerstaff et al. 1995). Additional research is required to understand how the improvements in respiratory measures might impact function post‐laryngectomy. A further limitation was the use of a single clinician for perceptual voice rating.

Few outcome measures exist which are specific to PWL; therefore, measures developed within laryngeal or other clinical populations were used without adequate validation. This study did not include measures of exercise tolerance, general health or performance status. This data may have been informative in exploring participants’ report of improved exercise tolerance. Furthermore, baseline assessment of health status could indicate which people have the greatest potential for response to EMST or identify a ceiling effect in healthier participants. This is recommended as a direction for future research. It was not within the scope of this study to investigate whether the use of EMST also had an effect on training skill or technique of breathing alongside targeting strength. Additionally, this study did not explore the potential of inspiratory muscle training or combination training, which may have indications for use post‐laryngectomy. This would be a relevant direction for future research.

Swallowing function was not investigated during this study; however, two participants gave qualitative feedback on swallowing and considered whether the muscular engagement of EMST could assist neopharyngeal bolus transit. EMST has been found to impact positively on swallowing in laryngeal populations; therefore, it would be pertinent to investigate the impact of EMST on post‐laryngectomy swallow function.

## 5 Conclusion

EMST was found to be acceptable and feasible for PWL; however, device adaptations were required for safe usage. Specifically, in relation to the prompt removal of the device from the neck stoma to prevent airway obstruction. This study was of a small scale but provided important early data to inform a larger‐scale study. Improvements were demonstrated post‐intervention in respiratory measures of MEP, FEV1, PF and in voice loudness, which aligned with participant‐reported measures of cough, self‐perception of communication and environmental and attitudinal impact. Further research is required in this area to generate a greater understanding of the role of respiratory muscle training in laryngectomy rehabilitation, and to target treatments more specifically to those who may benefit most from it.  Additionally, there is a need to develop new and existing assessments of tracheoesophageal voice to support reliable and valid measurement of clinical outcomes.

## Ethics Statement

This study received ethical approval from the City St George's, University of London Senate Research Ethics Committee (reference no. ETH2223‐1307).

## Consent

All participants provided informed written consent to take part in the study and for the dissemination of de‐identified data via publication.

## Conflicts of Interest

The authors declare no conflicts of interest.

## Supporting information




**Supplementary File**: jlcd70107‐sup‐0001‐SuppMat.docx

## Data Availability

The data that support the findings of this study are available from the corresponding author on request.
