# Supplementary material for: Expiratory Muscle Strength Training to Improve Voice and Respiratory Outcomes After Laryngectomy: A Feasibility Study
Source: Int J Lang Commun Disord. 2025 Aug 15;60(5):e70107. doi: 10.1111/1460-6984.70107 (PMC12357018; doi:10.1111/1460-6984.70107)
Supplement: Supplementary file 1 — Supplementary File: jlcd70107‐sup‐0001‐SuppMat.docx [file JLCD-60-0-s001.docx]

**Supplementary material:**

**Tidier checklist**

| **Item no.** | **Item** | **Description** |
| --- | --- | --- |
| 1. | **BRIEF NAME:**  Provide the name that describes the intervention | Expiratory Muscle Strength Training (EMST) |
| 2. | **WHY:**  Describe any rationale, theory or goal of the elements essential to the intervention | Targetting expiratory muscle engagement through training will increase expiratory muscle strength. In turn this will improve cough strength and facilitate mucus clearance*.*  Based on previous research it is hypothesised this may also increase volume and perceptual quality of tracheoesophageal voice. |
| 3. | **WHAT:**  Materials: describe any physical or informational materials used in the intervention, including those provided to participants or used in intervention delivery or training of intervention providers | An EMST device is required, which is a hand-held threshold pressure device. Participants will also require strongly adhesive baseplate and materials such as elastic bands to improve device airseal at the neck stoma.  Participants will provided with written information on how to use and clean the EMST device, a practice tracking diary and written space to capture reflections on practice. |
| 4. | **WHAT:**  Procedures: describe each of the procedures, activities and/or processes used in the intervention including enabling or support activities | The intervention requires participants to complete 25 exhalations per day into the device, for five days per week.  This will be carried out for five weeks.  Participants will then complete a six-week maintenance period of 25 breaths per day for two days per week.  Each week of the initial five-week intervention, participants will attend a check-in appointment with the research team to discuss the intervention, monitor challenges and progress, and to adjust the device when required. |
| 5. | **WHO PROVIDED:**  Describe expertise, background and any specific training given | SLTs with specialism in laryngectomy rehabilitation and voice therapy |
| 6. | **HOW:**  Describe the modes of delivery and whether provided individually or as a group | Two individual face-to-face therapy sessions to set up the device and implement safe practice, followed by weekly individual check-in sessions, which may be in person or via virtual contact according to participant preference. |
| 7. | **WHERE:**  Describe the type of locations where the intervention occurred | Small meeting room at City, University of London |
| 8. | **WHEN AND HOW MUCH:**  Describe the number of times the intervention was delivered, over what time period including number of sessions, schedule, duration, intensity or dose | The intervention was carried out for 25 repetitions per day, on five days per week, for five weeks.  Following the initial five-week intervention, participants completed a six-week maintenance period of 25 breaths per day for two days per week.  Participants were advised to complete the 25 daily breaths in five sets of five repetitions, spaced throughout the day according to their schedule.  The device threshold was set to 75% of maximal expiration pressure and adjusted according to gain in function at weekly check-ins. |
| 9. | **TAILORING:**  If the intervention was personalised, titrated or adapted; describe what, why, when, how | Participants could decide when to schedule the daily repetitions according to their normal daily routines.  The weekly check-ins were offered as face-to-face, online meeting, video-call or email/SMS chat. This was decided by participants according to their preference.  The threshold pressure setting of the device was set according to individual’s maximum expiratory pressure at baseline and adjusted as required. |
| 10. | **MODIFICATIONS:**  If the intervention was modified describe the changes | Device adaptations were made to support the use of the EMST device at the neck stoma |
| 11. | **HOW WELL:**  Planned: if intervention adherence or fidelity were assessed, describe how and by whom, and with what strategies | Adherance to the protocol was tested through the research team’s review of weekly practice diary, discussion with participants and through attendance data.  The research team followed a set protocol in delivering the intervention. |
| 12: | **HOW WELL:**  Actual: If intervention adherence or fidelity were assessed, describe the extent to which the intervention was delivered as planned | The intervention was delivered as planned.  There was 100% adherence to dosage in the five-week initial block, however, two participants completed this over 6/7 weeks.  Two participants reduced the dosage in the maintenance period. |
